# *CXCL12* methylation-mediated epigenetic regulation of gene expression in papillary thyroid carcinoma

**DOI:** 10.1038/srep44033

**Published:** 2017-03-08

**Authors:** Sijia Zhang, Yihan Wang, Meijun Chen, Lulu Sun, Jun Han, V. Kazakova Elena, Hong Qiao

**Affiliations:** 1 Department of Endoerinology and Metabolism, The Second Affiliated Hospital, Harbin Medical University, Harbin 150001, China; 2 College of Bioinformatics Science and Technology, Harbin Medical University, Harbin, 150081, China

## Abstract

Papillary thyroid carcinoma (PTC) is the most common type of thyroid cancer, and its incidence rate is rapidly growing. It is necessary to understand the pathogenesis of PTC to develop effective diagnosis methods. Promoter methylation has been recognized to contribute to the alterations in gene expression observed in tumorigenesis. Our RNA-seq data identified 1191 differentially expressed mRNAs and 147 differentially expressed lncRNAs in PTC. Next, promoter methylation of these genes was detected by reduced representation bisulfite sequencing (RRBS) technology and comprehensively analyzed to identify differential methylation. In total, 14 genes (13 mRNAs and 1 lncRNA), in which methylation was intimately involved in regulating gene expression, were proposed as novel diagnostic biomarkers. To gain insights into the relationships among these 14 genes, a core co-function network was constructed based on co-expression, co-function and co-methylation data. Notably, *CXCL12* was identified as an essential gene in the network that was closely connected with the other genes. These data suggested that *CXCL12* down-regulation in PTC may be caused by promoter hypermethylation. Our study was the first to perform an RRBS analysis for PTC and suggested that *CXCL12* may contribute to PTC development by methylation-mediated epigenetic regulation of gene expression.

Thyroid carcinoma is the most common malignant tumor of the endocrine system, accounting for 90% of all endocrine tumors, and its incidence has dramatically increased over the last 30 years^1^. Papillary thyroid carcinoma (PTC) is the predominant pathological type of thyroid carcinoma, accounting for more than 80% of all thyroid cancers, 60% for adults^2^ and 100% for children^3^. Approximately 32% of postoperative patients relapse or have lymphatic metastasis; patients with lymphatic metastases have a postoperative recurrence rate of up to 38.5–58.8%^4^. PTC is a strongly latent disease, and the diagnostic time is relative long; thus, studying PTC pathogenesis is of great significance.

In recent years, the deeper understanding of PTC and the rapid development of molecular detection technology have allowed, analyses of PTC at the molecular level, which have provided essential information to our understanding of PTC pathogenesis. For example, Huang *et al*. were the first to use chip detection technology to analyze the expression of 12,000 transcripts, which revealed a new molecular marker of PTC^5^. Choi *et al*. found that the cell fate regulator *PROX1* was inactivated in PTC at the mRNA expression, and that restoring *PROX1* expression in thyroid cancer cells not only activated the Wnt/β-catenin pathway, but also regulated multiple other PTC-associated genes, indicating that *PROX1* reactivation is a potential therapeutic strategy^6^. Other studies have suggested that the specific expression of Runx family genes in PTC, especially *Runx2, Runx3*, exerts considerable influence on cancer initiation and progression^7^,^8^. Furthermore, PTC is also associated with various oncogene mutations, such as activating *BRAF* or *RAS* mutations, and chromosomal rearrangements involving *RET *9. Handkiewicz-Junak *et al*. found that the *BRAF*^*V600E*^ mutation is present in 40–70% of PTC patients^10^. This hyper-activating mutation leads to persistent MAPK signaling, which has been shown to be a tumor initiating event.

Along with these genetic factors, epigenetic plays an important role in carncinogenesis. For example, DNA methylation is known to result in the transcriptional inactivation of tumor suppressors in the early stages of cancer. To date, investigations into DNA methylation in PTC can be divided in two types: those focused on 1) specific candidate genes or genes in the same pathway, or 2) the detection of whole genome methylation levels. Lee *et al*. specifically studied changes in the methylation and expression of three genes (*DUSP4, DUSP6* and *SERPINA5*) in PTC and their effects on the MAPK pathway, and discovered that *SERPINA5* expression was regulated by DNA methylation^11^. Kikuchi *et al*. used Infinium HumanMethylation27 BeadChip to analyze genome-wide DNA methylation patterns in 14 thyroid carcinoma patients and 10 heathy controls^12^. Kikuchi *et al*. demonstrated that six genes were silenced in PTC by DNA hypermethylation and suggested that these could be potential biomarkers. Based on Illumina450K BeadChip, White *et al*. found 1226 differentially methylated loci, including genes that are known to regulate pathways involved in thyroid carcinogenesis, such as *PI3K, PTEN* and *P53*^13^. Currently, the Infinium HumanMethylation450 BeadChip is broadly used in genome-wide DNA methylation studies due to its low cost, but it has limited CpG coverage, with less than 15,000 genes^14^. However, reduced representation bisulfite sequencing (RRBS) not only has a higher sensitivity, meaning less DNA is needed to directly detect simple base, but also has a wider range and detects more loci. Here, we present the first epigenomic data using RRBS in PTC.

In this study, we analyzed the genome-wide transcriptomes and epigenomes of three PTC patients using RNA-seq and RRBS, respectively. We also compared differentially expressed mRNAs and lncRNAs in tumor tissues and normal tissues. We then investigated the mechanism causing these alterations, and demonstrated that differential methylation occurred in the promoter regions of several affected genes. Through further analysis, we obtained 14 genes whose expression was negatively regulated by methylation in PTC that could act as potential biomarkers. Finally, a core co-functional network was constructed based on expression, methylation and functional similarities between the 14 genes, and revealed the important roles of *CXCL12* in PTC. *CXCL12* may be a potential therapeutic target for PTC which provided guidance for clinical diagnose and therapy.

## Results

### Analysis of PTC transcriptome characteristics

We performed whole transcriptome sequencing (RNA-seq) on three pairs of matched PTC tumor tissues and matched adjacent normal tissues. A total of 53.6 million reads were generated from single-end sequencing using the Hiseq 2500 sequencing system, on average there were 8.9 million reads per sample with an average read length of 50 bp. Cufflinks was used to assemble probable transcripts and yielded 286,587 distinct transcriptional loci. Additionally, by comparing our merged transcriptome with known gene annotations, we discovered 30 novel putative long intergenic noncoding RNAs (lincRNAs; see Materials and Methods). The expression of all transcripts after Cuffnorm normalization were in agreement, with strong pairwise correlation (Pearson Correlation), especially among the three normal samples and three tumor tissues (Supplementary Figure S1A).

**Figure 1:**
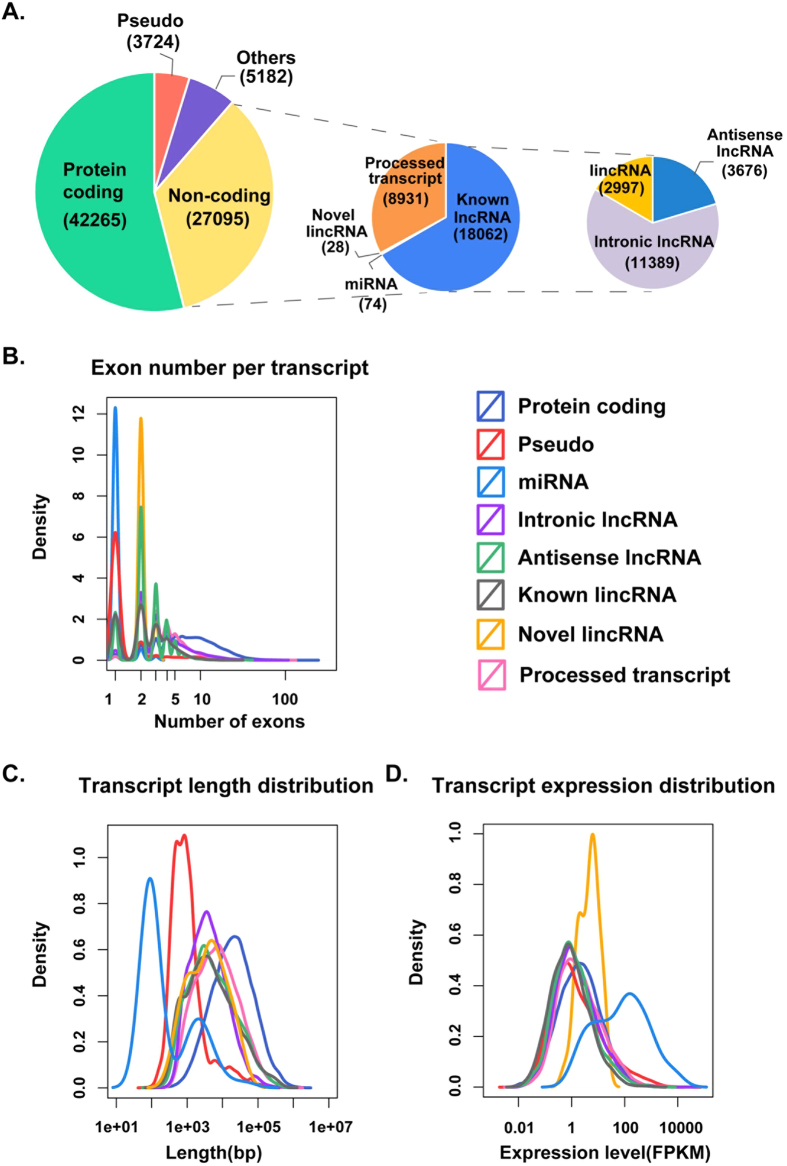
Analysis of transcriptome data in papillary thyroid carcinoma. (**A**) All individual transcriptomes were merged into a consensus transcriptome that included both tumor and normal tissues, and low expressed transcripts were discarded. The remaining transcripts were categorized as annotated protein-coding, non-coding, pseudo and other unannotated transcripts based on the Ensembl annotated genes. The novel lincRNAs we identified were included in non-coding RNAs. (**B**) The number of exons per transcript. (**C**) Transcript size distributions of different transcript types. (**D**) Transcript expression distributions of different transcript types.

The low expression transcripts with FPKM <0.01 in more than 50% of samples were removed from further study. Finally, the transcriptome assembly retained 78,266 distinct transcripts that were primary classified into 8 categories: protein-coding, pseudo, processed transcript, miRNA, antisense lncRNA, intronic lncRNA, known lincRNA and novel lincRNA (Fig. 1A). The analysis revealed that more than half (54%) of expressed transcripts were protein-coding transcripts (mRNAs) and non-coding RNAs accounted for approximately 35% of the total. Among the species of non-coding RNAs, we focused on the specific categories of long non-coding RNAs, such as novel lincRNAs, known lincRNAs, intronic lncRNAs and antisense lncRNAs. Both novel lincRNAs and known lncRNAs had fewer exons than protein-coding genes (2.14, 3.24 and 9.26 on average, respectively), likewise, they had shorter average transcript length (3932 bp, 4340 bp, and 19296 bp, respectively), while novel lincRNAs had higher expression than known lncRNAs and protein-coding genes (median 5.66 FPKM, 1.09 FPKM, and 1.96 FPKM, respectively) (Fig. 1B,C and D).

### Differentially expressed mRNAs and lncRNAs were functionally associated with PTC

**Figure 2:**
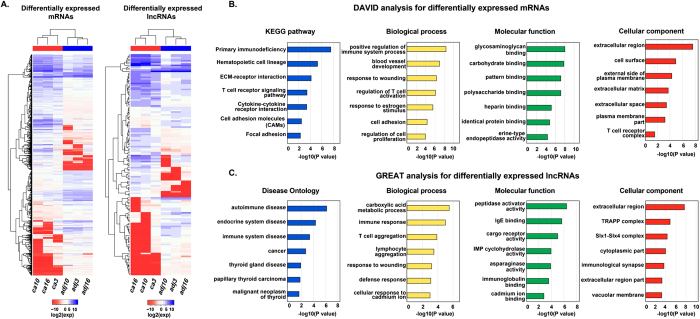
Hierarchical clustering and functional enrichment analysis for differentially expressed mRNAs and lncRNAs. (**A**) Two-way hierarchical clustering of differentially expressed mRNAs and lncRNAs in papillary thyroid carcinoma tissue samples and normal tissue samples. In the samples, “ca” and “adj” represent tumor and normal samples respectively, and “3”, “10”, “16” were the sample numbers. (**B**) KEGG pathway analysis and GO functional enrichment analysis for differentially mRNAs using DAVID. (**C**) Disease ontology analysis and GO functional enrichment analysis for differentially expressed lncRNAs using GREAT.

To characterize differentially expressed genes within our transcriptomes, we performed differential expression analyses for mRNAs and lncRNAs respectively. R package “edgeR” and fold change were combined to identify differentially expressed transcripts. In total, only 2597 mRNAs were differentially expressed in tumor samples compared with adjacent normal samples, including 935 up-regulated mRNAs and 1662 down-regulated mRNAs. The group of differentially expressed lncRNAs included 367 intronic lncRNAs, 161 antisense lncRNAs, 160 known lincRNAs and 6 novel lincRNAs, of which 281 were up-regulated and 413 were down-regulated. Hierarchical clustering analysis exhibited that samples from the same source were collapsed into one cluster, suggesting that the expression level of both differentially expressed mRNAs and lncRNAs in tumor tissues were significantly different from adjacent normal tissues. To validate our results, we also identified differentially expressed genes in The Cancer Genome Atlas RNAseq dataset. Among our differentially expressed genes, 601 up-regulated mRNAs, 590 down-regulated mRNAs, 77 up-regulated lncRNAs and 70 down-regulated lncRNAs also showed differential expression in this independent dataset. We used this overlapping set of more reliable differentially expressed genes for further analysis (Fig. 2A).

To explore the potential functions of the differentially expressed transcripts, we performed a functional enrichment analysis for the mRNAs using the DAVID functional annotation tool^15^. The results revealed that the differentially expressed protein-coding genes were significantly enriched for many PTC-associated pathways and biological functions, such as, ECM-receptor interaction, blood vessel development, response to wounding and regulation of cell proliferation (Fig. 2B).

One of the major challenges in studying lncRNAs is to determine their biological function; GREAT software provides a well-established way to assign biological meaning to non-coding regions by analyzing the annotations of the nearby genes^16^. The test set of 147 genomic regions picked 238 (1%) of all 18,041 genes. The disease ontology revealed that the differentially expressed lncRNAs were enriched in papillary thyroid carcinoma and associated diseases, and they were related to the biological processes of immune response and defense response (Fig. 2C).

### Methylome analysis of promoters with distinct CpG density

As DNA methylation is frequently altered in tumors, there is great interest in further exploring how these changes contribute to PTC and whether they affect transcription. To this end, DNA methylation profiles of the three matched pairs of PTC tumor tissues and adjacent normal tissues were also detected. The Reduced Representation Bisulfite Sequencing (RRBS) generated 359 million paired–end short reads, on average there were 59.9 million reads per sample with an average read length of 100 bp. After quality control using Trim Galore, 352 million clean reads were retained. The shared 1,036,077 CpG sites for each sample were used for further study. The pairwise correlation analysis revealed that all six samples had strong correlation (Supplementary Figure S1B).

**Figure 3:**
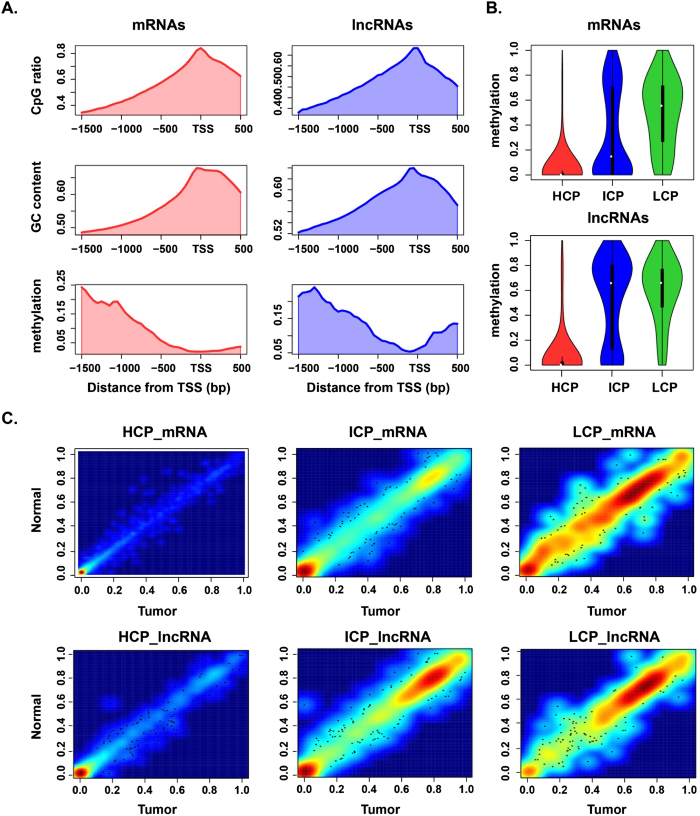
Methylation analysis of different CpG density promoters for mRNAs and lncRNAs. (**A**) The CpG ratio, GC content and methylation level at different distances from TSSs for all samples that included both tumor and normal tissues. (**B**) Boxplots of methylation levels in HCPs, ICPs and LCPs for all samples that included both tumor and normal tissues. (**C**) Scatterplots of methylation levels in HCPs, ICPs and LCPs for tumor and normal tissues, respectively.

Altered promoter methylation has been reported to be associated with various malignancies, in this study, we limited our analysis to CpG sites within promoter regions. Altogether, we found promoter methylation for 23,565 mRNAs and 7262 lncRNAs. As cytosine methylation can interfere with transcription factor binding via recruitment of methyl-CpG binding domain (MBD) proteins that induce chromatin changes^17^, the local density of CpG within promoters may affect the strength of repression. Therefore, we calculated the CpG ratio, GC content and methylation level in the promoter regions. The CpG density, including CpG ratio and GC content, were highest at transcription start sites (TSSs) and decreased beyond 500 bp from TSSs. Meanwhile, a negative relationship was found between methylation level around TSSs and CpG density (Fig. 3A). Therefore, we investigated the effect of promoter methylation on genes with different CpG density. Three classes of promoters were defined based on CpG ratio, GC content and length of CpG-rich region: high-CpG promoters (HCPs), low-CpG promoters (LCPs) and intermediate CpG promoters (ICPs)^18^. It was found that most promoters were HCPs (19,469, 82.62%), 3013 (12.79%) promoters were ICPs and 1083 (4.59%) ones were LCPs. The results exhibited that significant difference methylation patterns among the three classed of promoters. HCPs had the lowest methylation levels, whereas LCPs had the highest methylation levels, especially in mRNAs (Fig. 3B). Moreover, the methylation levels of mRNAs were higher than lncRNAs on the whole. Next, the methylation distribution patterns of tumor and normal tissues were shown in scatterplots, which implied that more hypomethylation (methylation level <0.2) in the ICPs and a bimodal distribution in the LCPs for mRNAs, while for lncRNAs there was a bimodal distribution in the ICPs and more hypermethylation (methylation level >0.6) in the LCPs (Fig. 3C).

### Identification of PTC-associated differentially methylated promoters

To explore whether promoter DNA methylation contributed to PTC, we identified differentially methylated mRNA and lncRNA promoter regions. We obtained 1182 differentially methylated mRNA promoters, of which 473 had higher methylation in tumors than normal tissues (hyper-DMPs) and 709 that had the opposite trend (hypo-DMPs). Smilarly, 364 lncRNA promoters were differentially methylated, including 166 hyper-DMPs and 198 hypo-DMPs. We performed a functional analysis of the differentially methylated mRNAs and lncRNAs using the same methods as for differentially expressed genes. The differentially methylated protein-coding genes were also involved in many cancer-related biological processes, such as cell adhesion, positive regulation of apoptosis, and positive regulation of programmed cell death. The significantly enriched terms of disease ontology for the differentially methylated lncRNAs included thyroid carcinoma, epithelial carcinoma and endocrine system disease.

**Figure 4:**
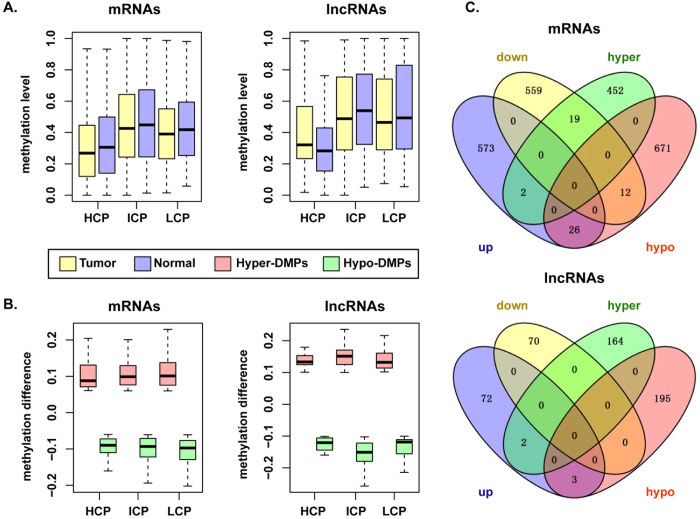
Analysis of differentially methylated promoters for mRNAs and lncRNAs. (**A**) Boxplots of methylation levels of different density DMPs in tumor and normal samples. (**B**) Boxplots of methylation differences at different density DMPs between tumor and normal samples. (**C**) Venn diagrams of differential expression and differential methylation.

In addition, Table 1 lists the number of hyper-DMPs and hypo-DMPs classified to the three classes of promoters. The results showed that differential methylation occurred more often in intermediate or low CpG density promoters for both mRNAs and lncRNAs. Notably, for lncRNAs, hypo-DMPs were enriched in ICPs (Fisher test, p = 6.767 × 10^−4^). To further characterize the features of the differentially methylated promoters, we displayed the methylation states among the three classes of promoters. The ICPs and LCPs had higher methylation levels than HCPs for both mRNAs and lncRNAs. However, for mRNAs, the difference values between tumor and normal were larger in the LCPs than HCPs and ICPs, and for lncRNAs, methylation in the ICPs showed the great difference (Fig. 4A and B).

### Differentially expressed genes regulated by promoter DNA methylation in PTC

It is widely accepted that promoter hypermethylation causes a downregulation of gene expression, whereas promoter hypomethylation is associated with the upregulation of gene expression^19^,^20^. To understand if changes in promoter methylation affect gene transcription in PTC, we evaluated groups of genes that showed both differential methylation and expression between tumor and normal tissues. Nineteen mRNAs were hypomethylated at their promoters and up-regulated in PTC and 26 were hypermethylated at their promoters and down-regulated, while only 3 lncRNAs showed hypermethylation and down-regulation (Fig. 4C). Thus, it seemed that promoter methylation of mRNAs may play more important roles in PTC.

**Figure 5:**
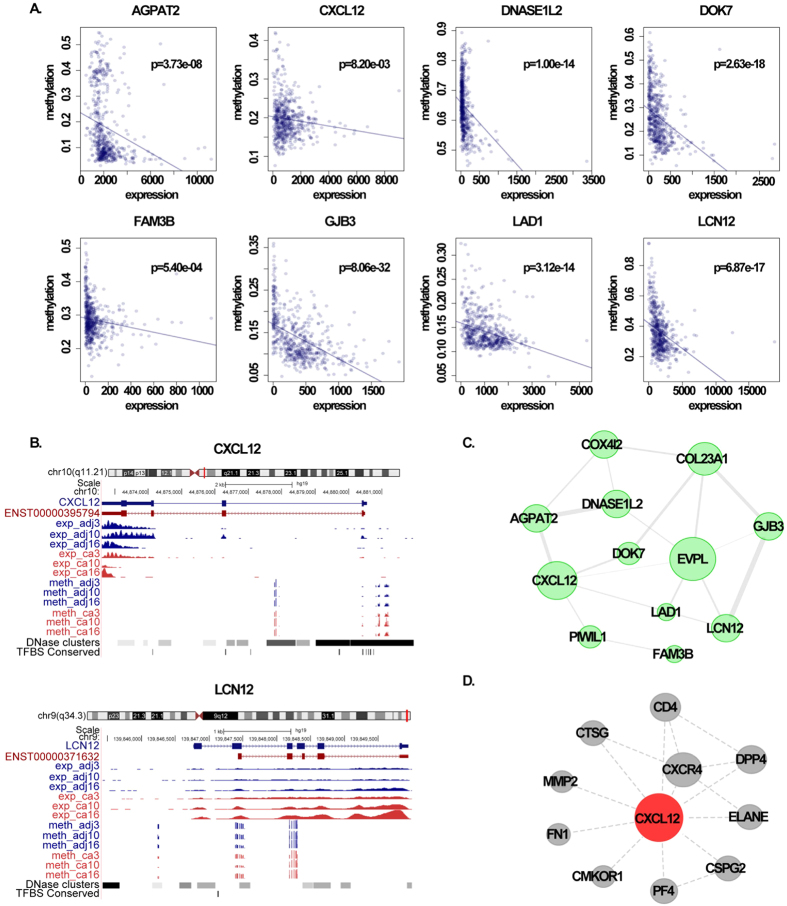
Visualization of methylation and expression of partial potential biomarkers. (**A**) Scatterplots show the significantly negative correlation between methylation and expression of eight genes using TCGA tumor datasets. (**B**) Examples were presented by UCSC using our RNA-seq and RRBS data. (**C**) The core co-expression network of 14 potential biomarkers. Nodes represent genes, and lines represent significant co-expression, co-function and co-methylation between gene pairs. (**D**) The *CXCL12* module extracted from the protein-protein interaction network.

Promoter CpG islands are primarily involved in gene regulation, and approximately 70% of annotated gene promoters are associated with a CpG island^21^. Among the 45 mRNAs and three lncRNAs, 13 mRNAs and one lncRNA that mapped to 14 individual genes had CpG islands in their promoters. We assumed that the differential methylation affected the transcription of these 14 genes, which included *CXCL12, FBLN7, FAM3B, PROX1, COL23A1, GJB3, LAD1, PIWIL1, DNASE1L2, EVPL, COX4I2, LCN12, AGPAT2* and *DOK7* (Table 2). As CpG islands were contained within their promoters, most of 14 genes were of high CpG density, except for *FAM3B, COL23A, COX4I2, LCN12* and *DOK7*, which were of intermediate CpG density. Receiver operating characteristic (ROC) curves and the area under the curve (AUC) were used to evaluate the diagnostic effects of these potential biomarkers and to determine appropriate cut-off points (Supplementary Figures S2, S3 and S4). The results showed that they had the ability to discriminate between the diseased and healthy populations. The AUCs of all genes were over 0.75 in at least two datasets. As the dataset GSE33630 were derived from microarray, there may be some bias from the RNA-seq data. Furthermore, we calculated the correlation between promoter methylation and gene expression for the 14 genes using HumanMethylation450 BeadChip and RNA-seq data from TCGA. The expression of *AGPAT2, CXCL12, DNASE1L2, DOK7, FAM3B, GJB3, LAD1* and *LCN12* were negatively correlated with methylation in patients (Pearson Correlation, p < 0.05, Fig. 5A and B). These results indicated that the differentially expressed gene associated with PTC were regulated by promoter methylation.

### Identification of potential biomarkers by constructing a core co-functional network

In light of the complexity in gene regulation, we expected to find links between DNA methylation and protein expression in PTC-associated genes. On the basis of our results, correlations between expression, methylation and semantic similarities of GO terms for the 14 genes were used to construct a core co-function network (Fig. 5C). The gene pairs which had significant expression, methylation and functional correlation were reserved and the Pearson correlation coefficients of methylation were used as linkage weights. Finally, we obtained a core co-function network based on methylation and expression among the 14 genes. Based on topological analysis of this network, we found *EVPL* and *CXCL12* had higher degrees than the others, reflecting their predominant roles. Meanwhile, in comparison to *EVPL, CXCL12* was found to be down-regulated and hypermethylation in both our data and TCGA datasets. Therefore, we speculated *CXCL12* might be involved in PTC carcinogenesis and could possibly serve as a diagnostic biomarker. C-X-C motif chemokine ligand 12(*CXCL12*), also termed stromal cell-derived factor 1(*SDF-1*), is a member of the CXC chemokine subfamily that plays a role in immune surveillance, inflammation response, tumor growth and metastasis. It has been reported that differential expression of *CXCL12* occurrs in numerous cancers. In a study of gastric cancer, Zhi *et al*. found that aberrant *CXCL12* methylation frequently causes a down-regulation of *CXCL12* expression and suggested that *CXCL12* may play a role in cancer progression and metastasis^22^. Researchers have also found that the constitutive expression of *CXCL12* in the colonic epithelium is silenced by DNA hypermethylation in primary colorectal carcinomas as well as colorectal carcinoma-derived cell lines^23^.

Furthermore, to explore *CXCL12* functions, a module was extracted from a protein-protein interaction network, which was integrated from five databases including HPRD, IntAct, DIP, MINT and BIND (Fig. 5D). The module contained genes directly connected with *CXCL12* and all the links within them. We noticed that 11 genes were contained in the module and they were enriched significantly in cell migration and cell motility, of which *CXCR4, DPP4* and *FN1* were also differentially expressed in our data. The contribution of the *CXCL12/CXCR4/CXCR7* axis to cancer progression has been increasingly recognized^24^. *CXCL12* is associated with the *CXCR4*-mediated activation of G protein-coupled signaling molecules, including ERK1/2, MAPK, JNK and AKT^25^,^26^. The *CXCL12/CXCR4/CXCR7* axis may contribute to thyroid cancer development by regulating cancer cell migration and invasion via AKT, ERK signaling and MMP-2 activation^27^. Furthermore Zhu *et al*. found that more than 90% of PTCs were associated with *CXCL12* immunohistochemical staining, indicating that *CXCL12* may be an effective supplementary diagnostic marker for PTC^27^.

## Discussion

In this study, by sequencing the whole genome DNA methylation and transcripts expression of the tumor tissues and normal thyroid tissues from three PTC patients, we performed an integrated analysis to investigate connections between epigenetic regulation and PTC pathogenesis. The aim was to discovery novel molecular diagnostic biomarkers that may be useful for early diagnosis and targets for future PTC therapies.

The rapid and persistently increasing incidence of PTC has made this disease a public health problem. With the increasing availability and applications of high-throughput sequencing methods, more and more studies have focused on the molecular mechanisms of cancer. Transcriptome analysis is an efficient tool for characterizing and understanding the molecular basis of phenotypic variation in cancers. Searching for differentially expressed genes is the most common analysis in transcription profiling. Clarifying the key genes in cancer-associated molecular events has great significance for PTC diagnosis and treatment. Here, we identified 1191 differentially expressed mRNAs and 147 differentially expressed lncRNAs, and found that they were involved in cancer-associated pathways and functions. For example, differentially expressed genes were enriched in the GO term extracellular matrix. Collagen is a major extracellular matrix component that plays a critical role in promoting tumor growth^28^. *COL23A1* is a transmembrane collagen that has been found to be differentially expressed in prostate and non-small-cell lung cancer^29^,^30^. Therefore, we inferred that the aberrant expression of *COL23A1* may also affect tumor development in papillary thyroid carcinoma.

Changes in environmental and lifestyle exposures are also factors that could lead to an increased risk of thyroid cancer incidence, but the specifics remain unclear^31^. Growing evidence has shown that aberrant epigenetic changes may participate in the regulation of gene expression and function^32^,^33^. DNA methylation is a cornerstone of epigenetics that has been widely studied in cancer. Aberrant DNA methylation- mediated gene expression alterations play a role in tumourigenesis and cancer progression, and has been reported in many carcinomas, including PTC^32^,^34^,^35^. Recently, novel sequencing technologies have been applied to tumor genomes and epigenomes, making comprehensive whole-genome detection of gene expression and DNA methylation more accessible, which has provided great insights into pathological mechanisms. However, only methylation arrays have been applied to the study of PTC, which limits the characterization of many potential risk loci and the representation of methylation patterns. Whole genome bisulfite sequencing is the gold standard for detecting DNA methylation, but its widespread application is limited by high sequencing costs^36^,^37^. Promoter methylation of 23,565 expressed mRNAs and 7262 expressed lncRNAs in PTC was described in this study. In addition, we were able to not only detect known differentially methylated genes, but also identified 14 novel genes regulated by DNA methylation in PTC. Our results also showed that the expression of lncRNAs may be less affected by promoter methylation in PTC.

To examine potential crosstalk between these genes, a core co-function network was constructed based on their correlation of expression, methylation and functional similarities. Within this network, we noted the key position of *CXCL12*, and further investigated interaction information using a protein–protein interaction network. In a small module consisting of *CXCL12, CD4, CXCR4* and *DPP4,* we found that three genes were differentially expressed, not *CD4*. A study has reported that *DPP4* inhibition can recruit regenerative stem cells via *CXCL12*, which infulenced ischaemia-reperfusion injury in murine lung transplantation model^38^. The *CXCL12–CXCR4* pair was found to be related to various tumors, including many solid cancers and hematopoietic malignancies^39^,^40^. Zhi *et al*. have proposed that loss of *CXCL12* and maintenance of *CXCR4* expression imparts metastatic cancer cells a phenotype similar to highly migratory circulating leukocytes and lymphocytes^22^,^41^. Studies have also reported that epigenetic down-regulation of CXCL12 is involved in breast carcinoma and non-small cell lung cancer metastasis^42^,^43^. In addition, *CXCL12* hypermethylation has been shown to be associated with lymph node metastasis development and higher proliferation rates of breast cancer cells^44^. Although previous studies have shown *CXCL12* to be a marker for many cancers including PTC, we propose that the essential role of *CXCL12* methylation is in regulating gene expression in PTC.

In conclusion, we investigated the potential biological significance of DNA methylation in PTC by integrating methylation and expression data, which revealed that the aberrant methylation of *CXCL12* was involved in carcinogenesis. This may contribute to the design of early diagnostic methods and/or future clinical trials for adjuvant chemotherapy for PTC patients.

## Materials and Methods

### Sample collection

This study was approved by the Ethics Committee of the Second Affiliated Hospital of Harbin Medical University, and written informed consent was obtained from all participants prior to inclusion. We obtained three matched pairs of pathologically-confirmed post-operative PTC tumor samples and normal thyroid tissues from three patients who underwent thyroidectomy at the Second Affiliated Hospital of Harbin Medical University. All methods were carried out in accordance with the relevant guidelines of the Ethics Committee of the Second Affiliated Hospital of Harbin Medical University. The six samples were immediately frozen with liquid nitrogen and stored at −80 °C after surgery. The ages of the patients, who were all female, were 37-, 39- and 41-year-old. The patients we recruited had not received preoperative radiotherapy or chemotherapy, and the specimens were not necrotic tissue. The controls were normal thyroid tissues more than 2 cm away from the tumor and without cancer cell infiltration.

### RNA-seq library preparation, sequencing and data processing

Tissues were thoroughly ground in liquid nitrogen, and 50–100 mg was added to 1 mL TRIzol and homogenized. The homogenate was then placed at room temperature for 5 min, centrifuged at 12,000 × *g* for 10 min at 4 °C, following which, supernatants containing RNA were collected. Then, samples were spun again at 4 °C at 12,000 × *g* for 15 min. The upper aqueous layer with RNA was obtained, and we precipitated RNA with isopropanol for 30 min, and collected after centrifugation at 12,000 × *g* for 10 min at 4 °C. RNA pellets were visible, and the supernatant was removed. The RNA precipitate was washed with 75% ethanol, dissolved in 25–200 μl RNase-free water and stored at −70 °C. A NanoDrop (Thermo Fisher Scientific, Waltham, MA, USA) was used for RNA quantitation. According to these results, we took 500 ng for 1% agarose gel electrophoresis. Then, we synthesized dscDNA, complemented the ends and added A-tailing buffer and processing joints. PCR enrichment and Qubit Instruments (Invitrogen Carlsbad, CA, USA) quantitative library were performed. The expression data was obtained using Illumina Hiseq2500 sequencing.

The libraries were sequenced using Hiseq2500 platform, and 50 bp single-end reads were generated. T Quality control of the raw reads was performed using FastQC, and then were mapped to the human genome (GCRh37/hg19) using Tophat v2.0.6^45^ with default parameters, except that the Gene Transfer Format (GTF) file for reads mapping in “-G” option were collected from Ensembl gene annotation. The mapped reads were assembled into transcripts guided by Ensembl gene models using Cufflinks v2.2.1^46^. All transcripts for the six samples were merged with Cuffmerge to generate a consensus transcriptome and their expression abundances were quantified by Cuffquant. To remove sources of bias in the data, the expression level of all transcripts were then normalized by Cuffnorm using the default normalization method. The low expressed transcripts with FPKM <0.01 in more than 50% of samples were filtered out.

### RRBS library preparation, sequencing and data processing

Tissues (not exceeding 25 mg) were ground in a 1.5 mL centrifuge tube, 200 μl ALT was added, mixed by vortexing, and after adding 20 μL proteinase K, were incubated in a 56 °C water bath for 30 min. Then, the samples were vortexed for 15s after adding 4 μL Rnase A and incubating at room temperature for 2 min. Then, vortexed for 15s after the addition of 200 μL AL and incubated in a 70 °C water bath for 10 min. We then added 200 μL of ethanol and put it into a column after mixing. After centrifugation at 6000 × g for 1 min, the filtrate was discarded. We then added 500 μL AW1, centrifuged at 6000 × g for 1 min and discarded the filtrate, added 500 μLAW2, centrifuged for 3 min at 20,000 × g and the collection tube and filtrate was discarded. The column was placed in a new 2 mL collection tube and centrifuged for 1 min at 20,000 × g. The column was then placed in a 1.5 mL centrifuge tube for 2 min at room temperature. The prepared tube was then placed into another 1.5 mL centrifuge tube and we added 80–200 μL ddH2O into the center of the membrane and incubated at room temperature for 2 min. Genomic DNA was eluted after centrifugation at 12,000 × g for 1 min. Quantifications were performed using a NanoDrop (Thermo Fisher Scientific). According to these results, we used 50 ng for 1% agarose gel electrophoresis, and 5 μg was subsequently fragmented, termini were complemented and A-tailing buffer and processing joints were added. Finally, 150–175 or 175–225 bp fragments were screened by 2% agarose gel electrophoresis and DNA was reclaimed using QIAGEN gel extraction kits (Hilden, Germany) according to the manufacturer’s recommendations. PCR enrichment and Qubit Instruments (Invitrogen, Carlsbad, CA, USA) quantitative library were performed. The methylation data was obtained using Illumina Hiseq2500 sequencing.

The libraries were sequenced using a Hiseq2500 platform, and 100 bp pair-end reads were generated. First, the raw low-quality reads were filtered and 3′/5′ adapters were removed using Trim Galore. Then, the clean reads were aligned to Ensembl human GCRh37/hg19 reference genome using Bismark v0.7.0^47^. Methylation calling of each CpG was processed by a module in Bismark called “Methylation Extractor”. From this analysis, 4–9 × 10^6^ CpG sites were detected in each sample, with an average sequencing depth of each sample in 11–27x. Only the CpG sites that were covered at least 5x in one sample were retained in the analysis. After removing the X and Y chromosomal loci, the number of the loci entered to the analysis was 1,036,077 CpG sites for each sample.

### Discovery of novel putative lincRNA transcripts

All available samples (tumor and adjacent normal) were used to discovery novel putative lincRNA transcripts. Firstly, we aggregated known gene annotations in gene transfer format (GTF) from Ensembl, RefSeq, ENCODE and UCSC, and removed redundancies. Additionally, lincRNAs identified from the Human Body Map project across 22 human tissues and cell lines were also downloaded from UCSC. Then, Cuffcompare was used to compare our merged transcriptome to a comprehensive list of known mRNAs and lncRNAs. Only transcripts over 200 nt with more than one exon and annotated by “x” were retained. We also removed transcripts that overlapped with known transcripts or were within 1000 bp of the nearest coding genes. Finally, to obtain reliable novel lincRNAs, the coding potential of putative lincRNAs were calculated using CPC^48^ and CPAT^49^, two robust approaches for distinguishing coding from noncoding RNAs. Only those transcripts with a CPC score <0 and a CPAT score <0.364 were retained as novel putative lincRNAs for the analysis.

### Identification of differentially expressed transcripts

The R package “edgeR”^50^ was used to identify differentially expressed transcripts between tumor and adjacent normal samples. Those transcripts with p values <0.05 and fold change >1.5(or <2/3) for each of the three pairs were defined as differentially expressed transcripts. To make our results more reliable, we downloaded thyroid cancer RNA-seq V2 isoform expression profiles of 513 tumor samples and 59 normal samples from TCGA and performed differential expression analyses using the same criteria.

### Definition of promoter classes

The promoter of a transcript was defined as 1500 bp upstream of the TSS to 500 bp downstream of the TSS. The promoters were divided into three classes^18^ according to CpG ratio, GC content and length of CpG-rich region, and included HCP, LCP and ICP. The CpG ratio was calculated as: (number of CpGs × number of bp)/(number of Cs × number of Gs). The three categories of promoters were determined as follows: HCPs contained at least a 500-bp window with a CpG ratio above 0.75 and GC content above 55%; LCPs do not contain a 500-bp window with a CpG ratio above 0.48; and ICPs are neither HCPs nor LCPs.

### Identification of differentially DNA methylated promoters

We calculated the average methylation level of all CpG sites located in the promoter region of a particular gene as the promoter methylation. For promoters of mRNAs and lncRNAs, the threshold for defining differential methylation was defined respectively based on methylation distributions. Promoters with absolute methylation differences between tumor and normal that ranked in the top 5% were regarded as differentially methylated promoters.

### Functional enrichment analysis

The functional enrichment analysis for differentially expressed mRNAs was performed using DAVID function annotation tool, which includes KEGG pathway, biological process, molecular function and cellular component. GREAT software, which assigns biological meaning to non-coding regions by analyzing the annotations of nearby genes, was used to analyze the function of lncRNAs. The terms were significant enrichment if hypergeometric test or binomial test p < 0.05.

### Construction of a core co-function network

A core co-function network was constructed by considering three relationships among potential biomarkers in PTC. First, Pearson correlation coefficients of expression were calculated and only significantly correlated pairs were extracted by performing 1000 permutation tests (p < 0.05). Next, semantic similarity of annotated GO terms was used to evaluate the functional similarity between the extracted gene pairs. The R package “GOSemSim”^51^ was used for this implementation. The pairs that had average similarity scores in BP, CC and MF branch >0.2 were reserved as functionally related gene pairs. Then, we calculated Pearson correlation coefficients of methylation between gene pairs. The significantly correlated pairs were reserved (Pearson correlation, p < 0.05) and Pearson correlation coefficients of each gene pair were used as linkage weights. Finally, we obtained a core co-function network based on expression, function and methylation among the genes.

## Additional Information

**How to cite this article:** Zhang, S. *et al. CXCL12* methylation-mediated epigenetic regulation of gene expression in papillary thyroid carcinoma. *Sci. Rep.* **7**, 44033; doi: 10.1038/srep44033 (2017).

**Publisher's note:** Springer Nature remains neutral with regard to jurisdictional claims in published maps and institutional affiliations.

## Supplementary Material

Supplementary Information

## Figures and Tables

**Table 1:** The number of DMPs and total promoters with three different promoter CpG density.

	hyper-DMPs	hypo-DMPs	Total
mRNA_HCPs	132(0.68%)	229(1.18%)	19469
mRNA_ICPs	184(6.11%)	283(9.39%)	3013
mRNA_LCPs	157(14.50%)	197(18.19%)	1083
lncRNA_HCPs	55(1.19%)	23(0.50%)	4609
lncRNA_ICPs	57(3.24%)	104(5.91%)	1761
lncRNA_LCPs	54(6.05%)	71(7.96%)	892

**Table 2:** The information of methylation and expression of 14 genes.

Ensembl_id	Type	symbol	T_meth (ave ± sd)	N_meth (ave ± sd)	T_exp (ave ± sd)	N_exp (ave ± sd)
ENST00000395794	mRNA	*CXCL12*	0.195 ± 0.029	0.120 ± 0.020	10.71 ± 9.13	41.87 ± 26.35
ENST00000272559	mRNA	*FBLN7*	0.333 ± 0.027	0.267 ± 0.038	3.80 ± 1.05	12.53 ± 1.24
ENST00000398647	mRNA	*FAM3B*	0.289 ± 0.075	0.192 ± 0.178	3.12 ± 3.83	9.55 ± 4.44
ENST00000366958	mRNA	*PROX1*	0.067 ± 0.115	0.000 ± 0.000	0.09 ± 0.07	1.09 ± 0.13
ENST00000390654	mRNA	*COL23 A1*	0.442 ± 0.125	0.370 ± 0.080	0.00 ± 0.00	0.86 ± 1.21
ENST00000373362	mRNA	*GJB3*	0.250 ± 0.072	0.502 ± 0.143	13.09 ± 3.77	0.23 + 0.27
ENST00000367313	mRNA	*LAD1*	0.189 ± 0.053	0.310 ± 0.069	22.33 ± 10.25	2.50 ± 0.66
ENST00000245255	mRNA	*PIWIL1*	0.567 ± 0.328	0.665 ± 0.061	2.48 ± 3.45	0.10 ± 0.12
ENST00000382437	mRNA	*DNASE1L2*	0.305 ± 0.008	0.361 ± 0.005	26.43 ± 4.45	2.26 ± 0.73
ENST00000589231	mRNA	*EVPL*	0.641 ± 0.166	0.703 ± 0.036	94.73 ± 50.55	13.32 ± 12.56
ENST00000376075	mRNA	*COX4I2*	0.380 ± 0.225	0.574 ± 0.107	141.33 ± 151.69	24.85 ± 28.68
ENST00000371632	mRNA	*LCN12*	0.282 ± 0.120	0.411 ± 0.123	306 ± 181.73	38.46 ± 38.70
ENST00000371694	mRNA	*AGPAT2*	0.052 ± 0.017	0.121 ± 0.012	210.18 ± 177.80	14.07 ± 24.36
ENST00000515886	lncRNA	*DOK7*	0.087 ± 0.058	0.267 ± 0.040	7.57 ± 2.82	0.99 ± 0.15
