# Supplementary material for: CXCL12 methylation-mediated epigenetic regulation of gene expression in papillary thyroid carcinoma
Source: Sci Rep. 2017 Mar 8;7:44033. doi: 10.1038/srep44033 (PMC5356381; doi:10.1038/srep44033)
Supplement: Supplementary Information [file srep44033-s1.pdf]

# *CXCL12* methylation-mediated epigenetic regulation of gene expression in papillary thyroid carcinoma

**Sijia Zhang[1], Yihan Wang[2], Meijun Chen[1], Lulu Sun[1], Jun Han[1], V.Kazakova Elena[1], Hong Qiao[1]***

[1]Department of Endoerinology and Metabolism, The Second Affiliated Hospital, Harbin Medical University, Harbin 150001, China

[2]College of Bioinformatics Science and Technology, Harbin Medical University, Harbin, 150081, China

*To whom correspondence should be addressed. Tel/Fax: 08686297720; Email: qiaoh0823@sina.com

## Supplementary Figures

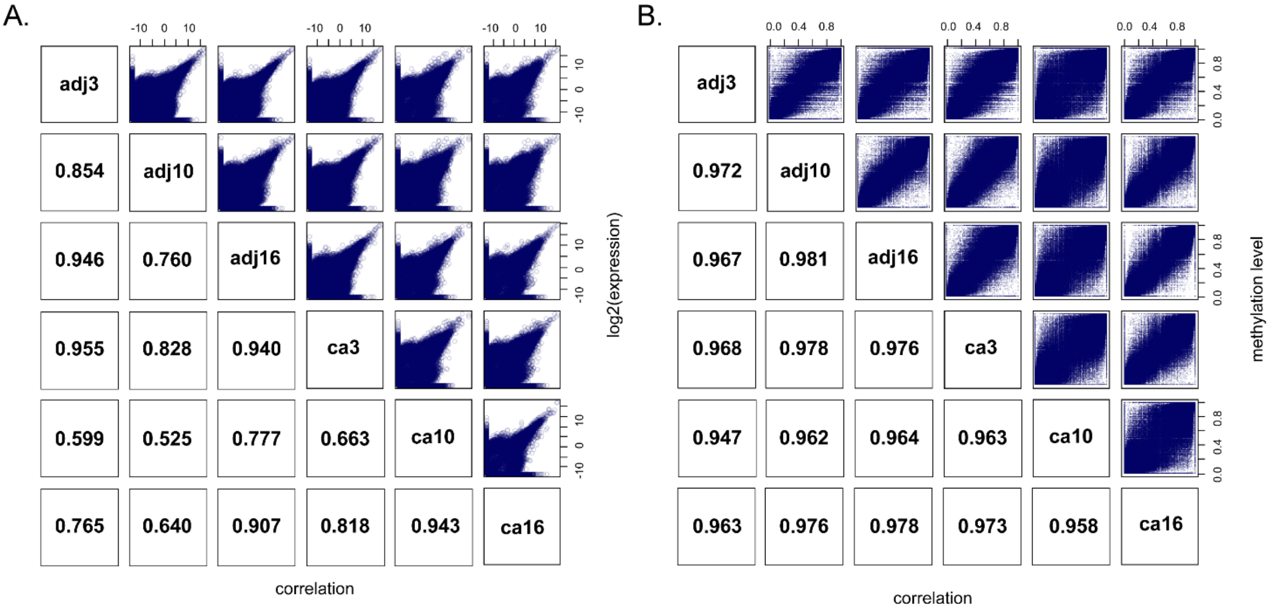


**Supplementary Figure S1. The correlation of transcript expression and DNA methylation between six samples.** "adj" represented the normal samples and "ca" represented the cancer samples. "3", "10, and "16" represented the number of the

patients. The Pearson correlation coefficients of all transcripts were calculated to evaluate the correlation between each two samples.

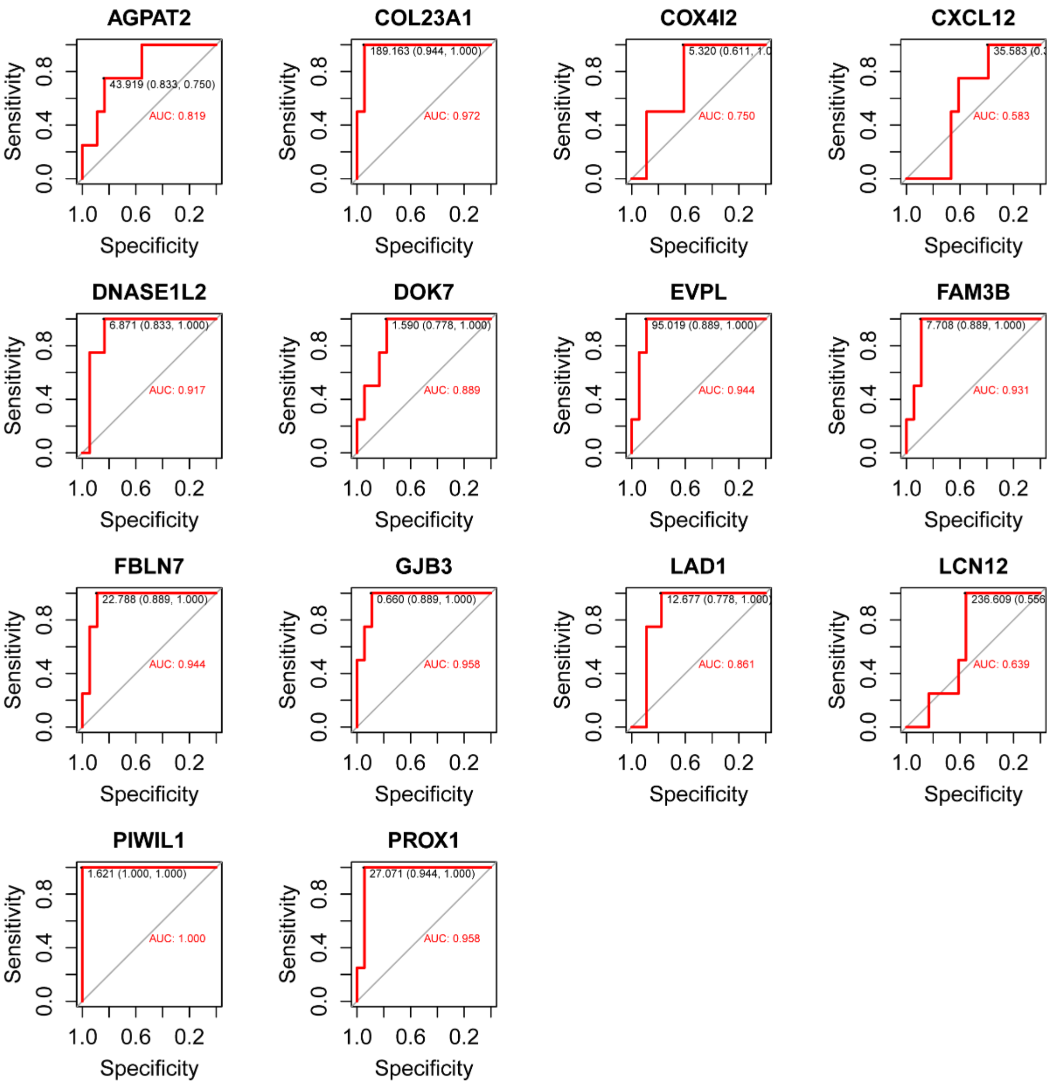


**Supplementary Figure S2.The ROC curves of the gene expression of 14 biomarkers using the data from GSE64912.**

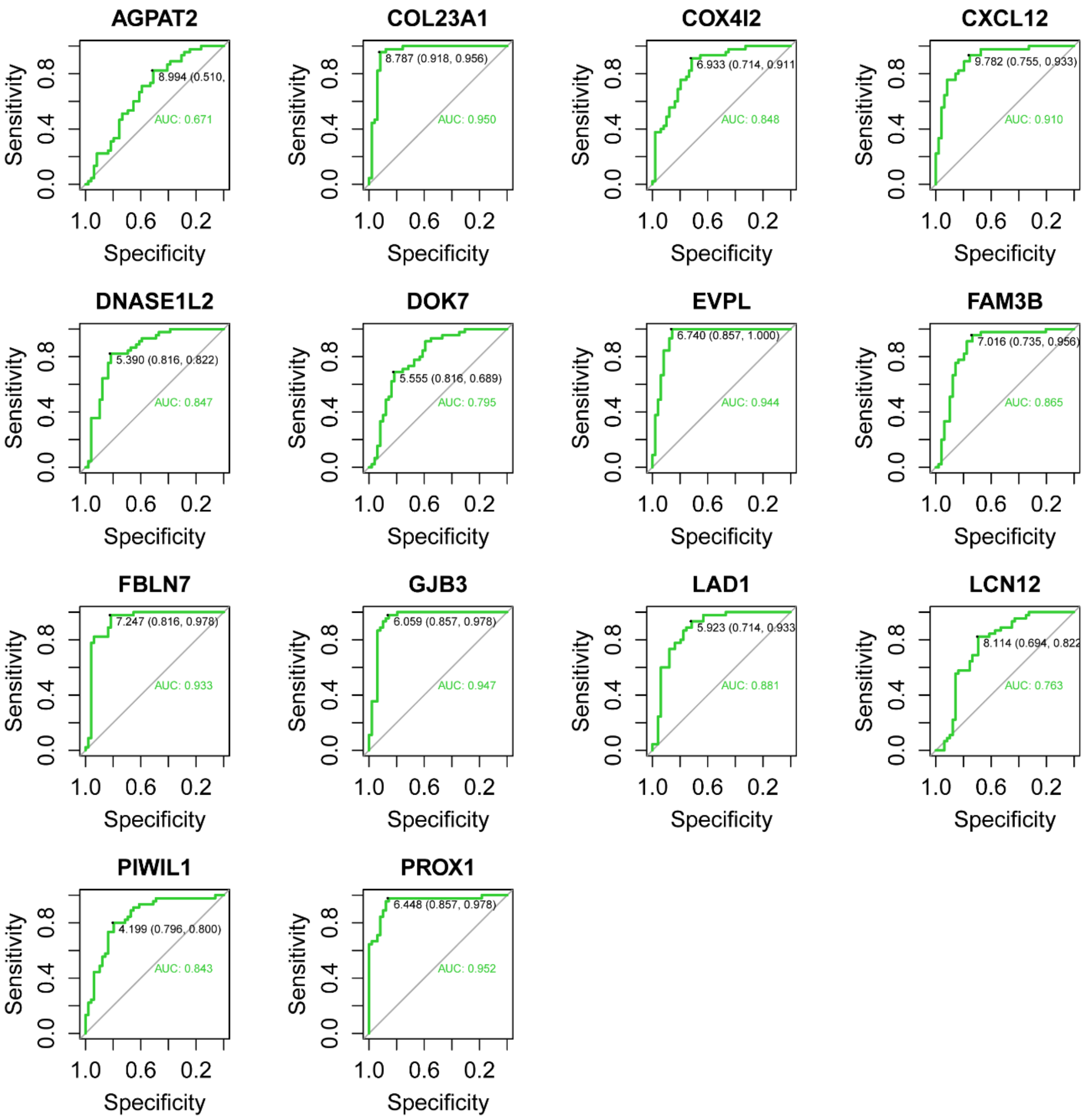


**Supplementary Figure S3. The ROC curves of the gene expression of 14 biomarkers using the data from GSE33630.**

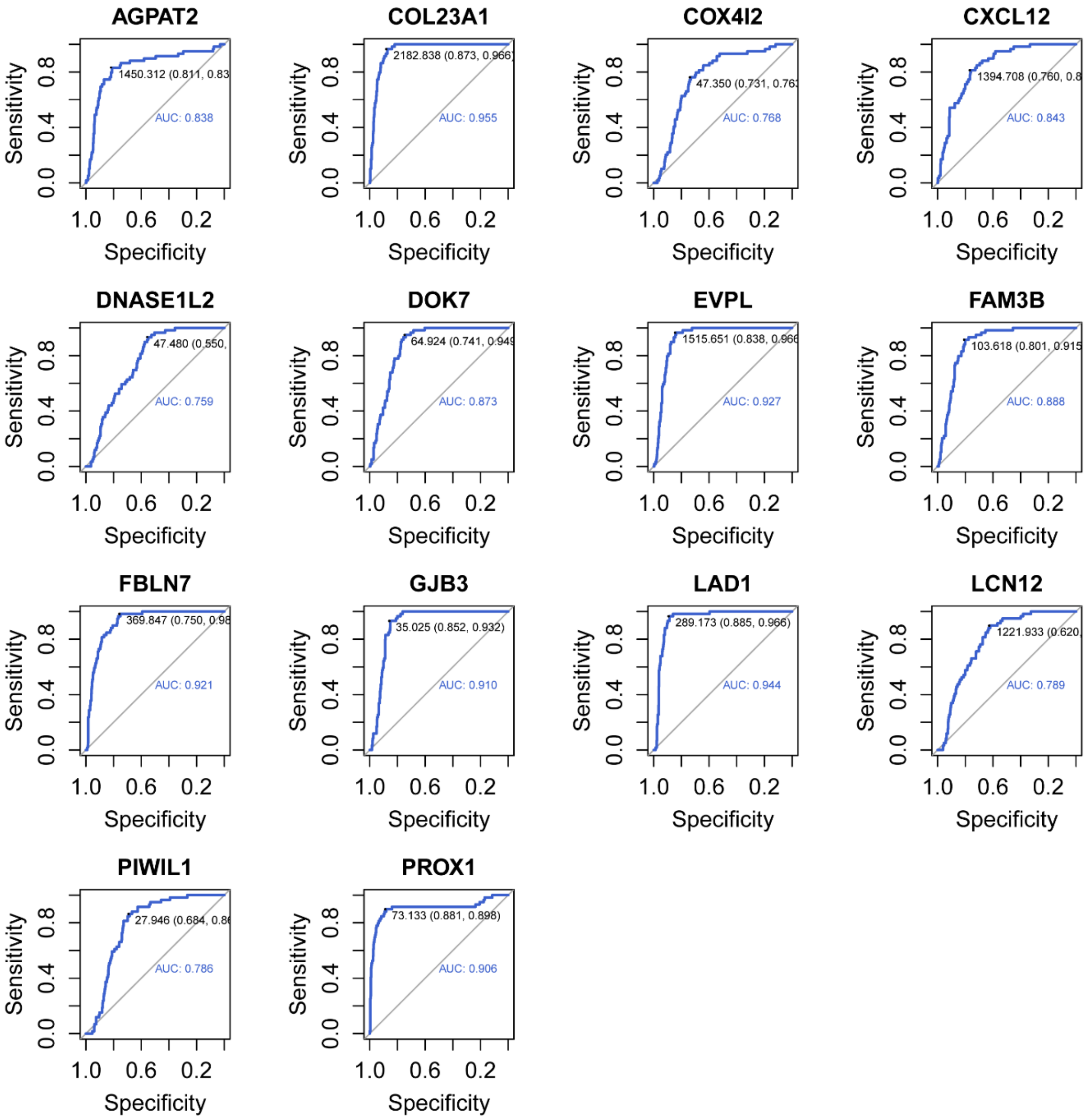


**Supplementary Figure S4. The ROC curves of the gene expression of 14 biomarkers using the data from TCGA RNA-seqV2.**
